# Dosimetric benefit of MR-guided online adaptive radiotherapy in different tumor entities: liver, lung, abdominal lymph nodes, pancreas and prostate

**DOI:** 10.1186/s13014-022-02021-6

**Published:** 2022-03-12

**Authors:** Lukas Nierer, Chukwuka Eze, Vanessa da Silva Mendes, Juliane Braun, Patrick Thum, Rieke von Bestenbostel, Christopher Kurz, Guillaume Landry, Michael Reiner, Maximilian Niyazi, Claus Belka, Stefanie Corradini

**Affiliations:** 1 Department of Radiation Oncology, University Hospital, LMU Munich, Marchioninistr. 15, 81377 Munich, Germany; 2 German Cancer Consortium (DKTK), Partner Site Munich, Munich, Germany

**Keywords:** Online MRI guided radiotherapy, Plan adaption, MRgOART, Online adaptive RT, MR-guided RT

## Abstract

**Background:**

Hybrid magnetic resonance (MR)-Linac systems have recently been introduced into clinical practice. The systems allow online adaption of the treatment plan with the aim of compensating for interfractional anatomical changes. The aim of this study was to evaluate the dose volume histogram (DVH)-based dosimetric benefits of online adaptive MR-guided radiotherapy (oMRgRT) across different tumor entities and to investigate which subgroup of plans improved the most from adaption.

**Methods:**

Fifty patients treated with oMRgRT for five different tumor entities (liver, lung, multiple abdominal lymph nodes, pancreas, and prostate) were included in this retrospective analysis. Various target volume (gross tumor volume GTV, clinical target volume CTV, and planning target volume PTV) and organs at risk (OAR) related DVH parameters were compared between the dose distributions before and after plan adaption.

**Results:**

All subgroups clearly benefited from online plan adaption in terms of improved PTV coverage. For the liver, lung and abdominal lymph nodes cases, a consistent improvement in GTV coverage was found, while many fractions of the prostate subgroup showed acceptable CTV coverage even before plan adaption. The largest median improvements in GTV near-minimum dose (D_98%_) were found for the liver (6.3%, *p* < 0.001), lung (3.9%, *p* < 0.001), and abdominal lymph nodes (6.8%, *p* < 0.001) subgroups. Regarding OAR sparing, the largest median OAR dose reduction during plan adaption was found for the pancreas subgroup (-87.0%). However, in the pancreas subgroup an optimal GTV coverage was not always achieved because sparing of OARs was prioritized.

**Conclusion:**

With online plan adaptation, it was possible to achieve significant improvements in target volume coverage and OAR sparing for various tumor entities and account for interfractional anatomical changes.

## Background

Various inter- and intra-fractional anatomical changes in patient anatomy pose a major challenge for the safe and successful treatment application in modern ablative image-guided radiotherapy (RT). Typical examples of such changes in patient geometry are different organ fillings of bladder, stomach or rectum, breathing-related motion, peristalsis, cardiac motion, tumor response (shrinkage), or organ and patient weight changes [1]. Numerous motion patterns of organs at risk (OAR), target volumes or quantification of motion amplitudes can be found in the literature [2–6].

These types of anatomical changes occur on various time-scales, ranging from seconds to weeks, and can potentially be accounted for via tumor-tracking or gating techniques and plan adaption strategies [1]. Although the technical implementation of such advanced RT techniques can be challenging, several adaptive RT (ART) approaches have found their way into clinical routine [1–7]. The feasibility and clinical benefit of offline ART using computed tomography (CT) or magnetic resonance imaging (MRI) has been demonstrated [8–12]. Strategies for online ART based on in-room cone-beam computed tomography (CBCT) have also been proposed [13–21]. A newly developed commercial CBCT-based system even allows for fast online ART in the clinical routine (Ethos™: Varian Medical Systems, Palo Alto, CA, USA) [22]. In this context, combined hybrid MR-Linac systems (e.g. MRIdian™: ViewRay Inc., Oakwood Village, USA; or Unity™: Elekta AB, Stockholm, Sweden) have the advantage of superior soft-tissue contrast and dose-free intrafractional imaging (where available, also with real-time tumor tracking and gated RT) in addition to a full online ART workflow. After the first patient was treated on such a hybrid device in 2017 [23], combined MR-guided RT systems for online ART (“MR-Linacs”) are now commercially available and becoming increasingly popular [24]. The feasibility of online magnetic resonance guided ART (oMRgRT) has already been demonstrated [25–31] and initial studies have reported its dosimetric and clinical benefits in a wide range of indications [32–46]. The aim of the present study was to evaluate the potential of treatment adaption in oMRgRT in terms of improved target volume coverage and OAR sparing across five different tumor entities, which are frequently treated with oMRgRT.

## Methods

### Patients

Overall, 50 patients treated between 01/2020 and 11/2021 were included in this retrospective analysis. All patients were treated for one out of five different tumor entities, which are typical indications for oMRgRT (liver, lung, abdominal lymph nodes, pancreas or prostate). Ten patients per entity were randomly selected from all patients of the respective subgroups, who successfully completed their RT treatment in the given period. Table 1 provides an overview of patient and treatment characteristics. All patients received hypofractionated oMRgRT using the MRIdian system (ViewRay Inc., Oakwood Village, OH, USA) with a step-and-shoot intensity modulated radiotherapy (IMRT) technique in the thoracic or abdominal region according to the institutional oMRgRT clinical protocol. The commercially available system consists of a hybrid MR-Linac and an integrated treatment planning system (TPS). Dose prescription referred to either the 65%, 80% or 95% isodose. Two patients were treated for two lesions simultaneously (patients 35 and 49) and one patient was treated for three lesions simultaneously (patient 42). In those three patients, the multiple lesions were treated with one single treatment plan. Thus, all OAR constraints of the corresponding fractions were counted as if these patients only had a single lesion. Parameters for target volumes (both gross tumor volume (GTV) and planning target volume (PTV)) were evaluated separately for each lesion. A total of 265 online adapted fractions were analyzed. Only fractions which were adapted were considered in the analysis.

**Table 1:** Patient characteristics

Patient Nr	Nr. of lesions	Nr. of adapted fx	Total nr. of fx	Fraction dose (Gy)	Total dose (Gy)	Prescription (%)	Group
1	1	3	20	3	60	95	Prostate
2	1	14	20	3	60	95	Prostate
3	1	5	5	7.25	36.25	95	Prostate
4	1	7	20	3	60	95	Prostate
5	1	20	20	3	60	95	Prostate
6	1	19	20	3	60	95	Prostate
7	1	20	20	3	60	95	Prostate
8	1	16	20	3	60	95	Prostate
9	1	9	20	3	60	95	Prostate
10	1	5	5	7	35	95	Prostate
11	1	2	5	8	40	95	Pancreas
12	1	4	5	8	40	95	Pancreas
13	1	5	5	8	40	80	Pancreas
14	1	5	5	8	40	80	Pancreas
15	1	5	5	8	40	80	Pancreas
16	1	5	5	8	40	80	Pancreas
17	1	5	5	8	40	80	Pancreas
18	1	5	5	8	40	80	Pancreas
19	1	5	5	6.6	33	80	Pancreas
20	1	5	5	8	40	80	Pancreas
21	1	3	3	12.5	37.5	65	Liver
22	1	3	3	12.5	37.5	65	Liver
23	1	3	3	12.5	37.5	65	Liver
24	1	3	3	12.5	37.5	65	Liver
25	1	3	3	15	45	65	Liver
26	1	3	3	12.5	37.5	65	Liver
27	1	2	3	15	45	65	Liver
28	1	3	3	12.5	37.5	65	Liver
29	1	3	3	15	45	65	Liver
30	1	2	3	12.5	37.5	65	Liver
31	1	5	5	7	35	95	Lymph nodes
32	1	4	5	5	25	80	Lymph nodes
33	1	2	5	8	40	95	Lymph nodes
34	1	4	5	6.4	32	80	Lymph nodes
35	2	5	6	6	36	80	Lymph nodes
36	1	9	10	4	40	95	Lymph nodes
37	1	5	5	7	35	95	Lymph nodes
38	1	3	5	7	35	95	Lymph nodes
39	1	4	5	7	35	80	Lymph nodes
40	1	5	5	6	30	80	Lymph nodes
41	1	3	5	10	50.0	95	Lung
42	3	3	3	13.5	40.5	65	Lung
43	1	2	3	13.5	40.5	65	Lung
44	1	3	3	13.5	40.5	65	Lung
45	1	3	3	13.5	40.5	65	Lung
46	1	2	3	13.5	40.5	65	Lung
47	1	3	3	13.5	40.5	65	Lung
48	1	3	3	13.5	40.5	65	Lung
49	2	3	3	13.5	40.5	65	Lung
50	1	2	3	13.5	40.5	65	Lung

The dose prescription refers to the corresponding isodose

The mean percentage of adapted fractions per patient was 86% (range 15% to 100%) and 79% of all fractions were adapted in total. Table 2 (Results) shows the portion of adapted plans and characteristics of the online adapted plans for each subgroup.

### oMRgRT workflow

The oMRgRT workflow was similar to that described by Bohoudi et al. [2]. For initial treatment planning, a planning MR and CT were acquired using the same patient setup. The planning CT was acquired immediately after the MR. The CT was registered using deformable image registration (DIR) to the MR to obtain electron density values for dose calculation. GTV, clinical target volume (CTV) and OAR delineation was performed on the MR. In the TPS, Boolean operations of regions of interest (ROIs; e.g. subtraction or margin expansion of structures) can be performed and stored as so-called “rules”. Such rules were defined for the automatic generation of the PTV (expansion of the GTV) and derived structures were defined at the treating physician’s discretion to reduce the contouring effort during online adaption. After dose prescription and contour delineation, a baseline treatment plan was generated analogous to the workflow in conventional RT. All plans were generated as step-and-shoot IMRT via inverse planning (6 MV flattening filter free beam; 1.5 mm calculation grid size with isotropic voxels; 1.0% Monte Carlo dose calculation uncertainty) and the maximum number of multi leaf collimator (MLC) segments was limited, depending on the complexity of the plan. This segment number limit of the baseline plan was subsequently used for online plan adaption. For each treatment fraction, a balanced steady-state free precession (bSSFP) pulse sequence 3D setup MRI scan was acquired for translational patient setup correction (couch shift). For more information about the MR pulse sequences and the technical design of the MRIdian system, refer to Klüter et al. [47]. The MRI of the baseline plan was then registered via DIR to the volumetric setup MRI of the day and all target structures, OARs and the electron density of the planning CT were propagated onto the setup MRI. All contours were edited (if necessary), a tracking contour was defined and the baseline plan was calculated on the MRI (more precisely the synthetic CT) of the day, which results in the so-called predicted dose (baseline plan calculated on the anatomy of the day with updated structures). This dose distribution shows the dose of a single non-adapted fraction and is the basis to decide whether to adapt a plan or not. In case of a subsequent plan adaption, the initial predicted dose corresponds to the dose distribution prior to plan adaption. For most OARs, a time-saving and practical partial re-contouring approach was used, in which the OARs were edited only in the close surrounding of the PTV (PTV + 3.0 cm), where the highest dose gradients occur. This approach was described by Bohoudi et al. [2], while Ahunbay et al. described a similar basic concept [48]. When the decision was made to adapt the plan of the current fraction, the plan parameters and dose constraints of the baseline plan were used as a starting point for dose optimization. A treatment plan was adapted if either the target coverage or OAR constraints of the predicted plan were not fulfilled, or a combination of both. Therefore, the planning goal was always to achieve optimal target coverage while respecting all OAR constraints. Online plan adaption was performed either as re-optimization with the same objectives of the baseline plan or as full re-optimization with modified objectives and/or plan parameters. The dose distribution of the online adapted plan, calculated on the current synthetic CT (based on the MR of the day) with updated structures is referred to as re-optimized dose. All dose calculation settings for the re-optimized dose were the same as for the predicted dose (as defined in the baseline plan). After plan adaption, the dose was verified for QA using a secondary Monte Carlo code before treatment.

For tumor tracking via a 2D bSSFP cine MRI sequence, the tracking structure was propagated onto a 2D cine MRI slice and a gating ROI was created by expansion of the tracking structure. These structures were subsequently used for online beam gating. All patients in which the target volume showed a breathing-related motion were treated using a breath-hold technique (mostly deep inspiration breath hold). However, patients treated with a free breathing approach (in cases of very limited tumor motion, e.g. most prostate cases) were also treated using the automated gating function in order to ensure that the target was positioned within tolerance boundaries during treatment application.

All baseline plans were validated dosimetrically with an ionization chamber and/or diode detector array (ArcCheck-MR; Sun Nuclear Corporation, Melbourne, FL, USA) prior to the first fraction.

### Extraction of DVH and plan parameters

Several dose volume histogram (DVH) parameters were extracted from the TPS for the predicted (non-adapted) and re-optimized scenarios for all fractions: the dose to 98%, 95%, 50% and 2% of the volume of the PTV (PTV D_98%_ = near minimum dose, PTV D_95%_, PTV D_50%_ = median dose, PTV D_2%_ = near maximum dose) and the mean PTV dose (PTV D_mean_). All parameters were also reported for the GTV. In prostate cases, the CTV was reported, as no GTV was defined. Furthermore, the volume of the PTV (V_PTV_) was extracted. Out of usually multiple, patient-specific OAR constraints prescribed in the TPS, three OAR constraints were chosen for OARs, which were closest to the PTV and as a result received the highest maximum dose values. Only OAR constraints were chosen, which were related to structures that were updated (in the PTV + 3.0 cm region; see last sub-section). The individual OAR dose constraints (near maximum dose or dose-to-volume constraints) depend on the dose prescription and the individual case and were defined by a senior physician based on the applicable guidelines.

Technical plan parameters like the net beam-on time (BOT), the number of segments, the number of Monitor Units (MU) and the number of beams were read out from treatment plan documentation files (Table 2; Results).

### Comparison of DVH parameters, statistical analysis and definition of dosimetric endpoints

First the OAR related DVH parameters for the predicted and re-optimized doses of each adapted fraction were compared.

Second, the same comparison was made for target volume (PTV, CTV or GTV) related DVH parameters. These parameters were systematically compared pairwise to quantify the DVH-based dosimetric benefit of online plan adaption for each subgroup separately. Primary dosimetric endpoints were chosen as follows: increase in GTV (all cases expect prostate) or CTV (prostate cases) D98%, increase in GTV or CTV D95%, increase in GTV mean dose, reduction in OAR exposure. The GTV (or CTV) near-minimum parameters (D98% and D95%) were chosen because underdose (e.g. dose drop at the surface or cold spots) within the GTV/CTV are likely to affect the local control (LC). The GTV (or CTV in prostate cases) mean dose was chosen because there is evidence for a predictive value of this parameter in terms of improved LC in liver [49] and lung tumors [50]. Boxplots were generated to visualize the data. Therefore, due to the different dose prescriptions, data was normalized. The D_98%_ and D_95%_ dose values were normalized to the prescribed dose (PD) for non-homogenous stereotactic prescriptions (65% or 80% prescription isodose PI) or 0.95 × PD for homogenous prescriptions (95% PI). The D_50%_ and D_mean_ dose values were normalized to the PD. The D_2%_ was normalized to PD/PI (thus PD/0.8 for a prescription to the 80% isodose) for stereotactic prescriptions or to PD for homogenous prescriptions. Since all normalization factors depend solely on the planning aim for the PTV, these factors may differ between patients but the same factors are applied to all fractions of a single patient. The dose normalization was done to achieve comparable data for the generation of analysis figures. Statistical analysis or the calculation of percent changes of dose values is not affected by this normalization. Statistical analysis of the DVH-based parameters was performed via paired Wilcoxon signed-rank test. A significance level of *α* = 5% was used.

## Results

Characteristics of the online adapted plans are shown in Table 2.

**Table 2:** Characteristics of the online adapted plans

	Liver	Lung	Lymph nodes	Pancreas	Prostate
Adapted fractions (%)	93.3	84.4	82.1	92.0	69.4
Mean BOT (min)	7.1	9.1	4.5	6.2	1.9
Min. BOT (min)	4.9	4.5	2.5	3.2	1.1
Max. BOT (min)	11.3	17.7	9.4	9.3	4.4
Mean number of beams	12	10	16	15	14
Min. number of beams	9	8	9	13	9
Max. number of beams	16	15	19	17	21
Mean number of segments	33	26	72	70	54
Min. number of segments	13	9	12	46	33
Max. number of segments	54	40	100	95	129
Mean MU	4269.2	5439.9	2652.2	3688.4	1139.4
Min. MU	2950.3	2675.2	444.3	1906.7	649.0
Max. MU	6776.0	10,612.0	5607.9	5547.1	2624.5
Mean V_PTV_ (cm^3^)	38.4	15.2	65.5	251.1	114.7
Min. V_PTV_ (cm^3^)	7.3	3.9	1.9	59.6	67.9
Max. V_PTV_ (cm^3^)	109.9	32.0	291.3	455.8	192.6

BOT = beam-on time, MU = number of monitor units, V_PTV_ = volume of the PTV

**Fig. 1:**
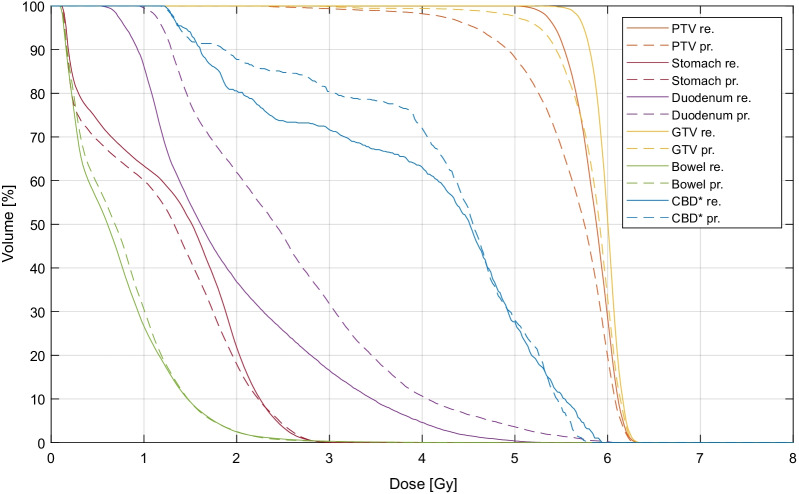
Exemplary cumulative DVH of fraction 2 of patient 32 under the adaptive workflow (abdominal lymph node treatment with a dose prescription of 5 × 5.0 Gy to the 80% isodose); re-optimized (re., solid lines) and predicted (pr., dashed lines). Target coverage increased after plan adaption while OAR exposure could be reduced. *Common bile duct

Figure 1 shows an example DVH of a single adaptive fraction of an abdominal lymph node case and illustrates the large potential of online ART to increase target coverage and OAR sparing with the oMRgRT technique. For example, the GTV D_95%_ improved by more than 10.0% and the duodenum D_50%_ was reduced by about one third.

**Fig. 2:**
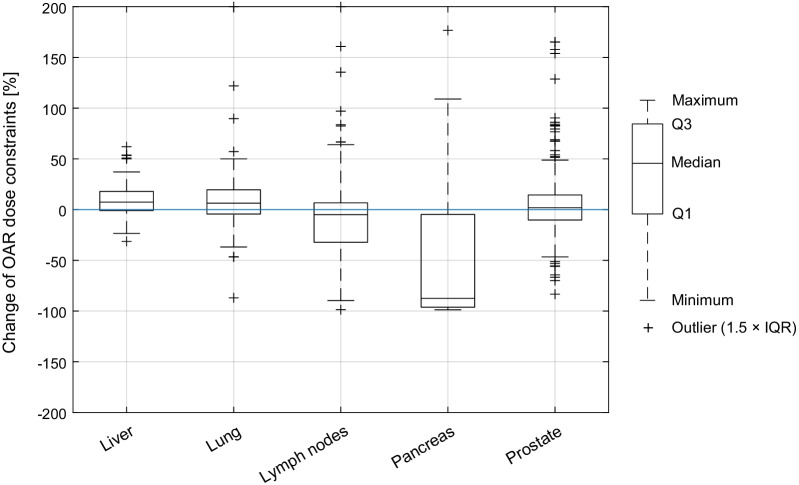
Boxplots of percent changes of OAR DVH parameters re-optimized versus predicted for all subgroups. The negative (lower) half corresponds to fractions where OAR exposure was reduced when adapted and the positive (upper) half corresponds to increased OAR exposure when adapted. Q1: first quartile, Q3: third quartile, IQR: interquartile range

Figure 2 indicates for each subgroup the change of OAR exposure when plans were adapted. The three most frequently considered OARs per region were bowel, duodenum, stomach (for liver), lung, heart, esophagus (for lung), bowel, duodenum, spinal cord (for lymph nodes), duodenum, stomach, bowel (for pancreas), and rectum, bladder, femur (for prostate). The largest median dose reduction of OARs adjacent or close to the PTV was found for the pancreas subgroup (-87.0%). This dose reduction was significant, as well as the smaller dose reduction achieved in the lymph-nodes subgroup. Small but significant median increased OAR doses were found for liver and lung and no statistically significant difference was found for prostate. All *p*-values and median percent changes are shown in the first row of Table 3. For a more detailed insight in the effect on individual OARs, Table 4 provides mean and median percent changes of OAR dose parameters of three most frequently considered OARs per region.

**Table 3:** *p*-values and median percent changes [*p* value/median change (%)] of DVH OAR and target volume parameters when comparing the re-optimized versus the predicted dose distributions

	Liver	Lung	Lymph nodes	Pancreas	Prostate
OAR	**0.000/6.9**	**0.004/5.9**	**0.001/−4.5**	**0.000/−87.0**	0.135/0.8
PTV D_mean_	**0.000/3.8**	**0.006/1.0**	**0.000/3.0**	**0.041/0.8**	**0.000/0.9**
PTV D_2%_	0.712/0.1	0.131/**−**0.7	0.757/0.0	**0.002/−0.9**	0.093/**−**0.2
PTV D_50%_	**0.001/2.6**	0.065/0.0	**0.003/0.9**	0.196/**−**0.5	**0.021/0.3**
PTV D_95%_	**0.000/17.3**	**0.000/6.0**	**0.000/9.4**	**0.000/5.7**	**0.000/2.8**
PTV D_98%_	**0.000/25.5**	**0.000/7.8**	**0.000/15.6**	**0.000/11.0**	**0.000/5.8**
GTV or CTV D_mean_*	**0.004/1.0**	**0.012/0.9**	**0.000/1.4**	0.454/**−**1.3	**0.007/0.4**
GTV or CTV D_2%_	0.872/0.0	**0.042/−0.4**	0.949/**−**0.2	0.176/**−**0.8	0.176/**−**0.1
GTV or CTV D_50%_	**0.040/0.6**	0.127/0.6	**0.020/1.0**	0.164/**−**1.4	0.185/0.2
GTV or CTV D_95%_	**0.001/4.6**	**0.000/3.1**	**0.000/4.9**	0.946/−0.3	**0.000/0.7**
GTV or CTV D_98%_	**0.000/6.3**	**0.000/3.9**	**0.000/6.8**	0.589/0.5	**0.000/1.3**

Significant differences are highlighted bold

*GTV for all subgroups except prostate and CTV for prostate

**Table 4:** Mean and median percent changes when comparing the re-optimized versus the predicted dose distributions [mean change (%)/median change (%)] of the most frequently used OAR dose parameter for each of the three most frequently considered OARs per region

Liver	D_max_ (bowel)	D_max_ (duodenum)	D_max_ (stomach)
	18.2/8.5	35.6/50.8	11.2/14.0

Lung	V15_Gy_ (lung left or right)	D_max_ (heart)	D_max_ (esophagus)
	9.0/5.9	18.3/0.0	3.6/8.2

Lymph nodes	V20_Gy_ (bowel)	V18_Gy_ (duodenum)	D_max_ (spinal canal)
	− 10.8/− 27.8	− 43.9/− 48.2	− 2.9/− 4.4

Pancreas	V33_Gy_ (duodenum)	V33_Gy_ (stomach)	V33_Gy_ (bowel)
	− 94.8/− 97.1	− 84.0/− 99.1	− 83.4/− 98.6

Prostate	V40_Gy_ (rectum)	V40_Gy_ (bladder)	D_max_ (femur left or right)
	6.8/0.3	9.3/7.4	− 0.7/− 1.2

**Fig. 3:**
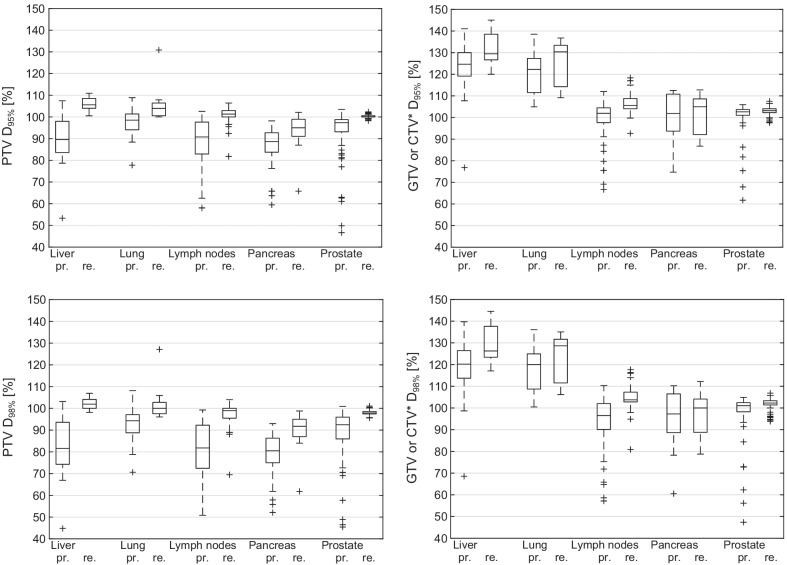
Boxplots for all subgroups of DVH target volume dose values D_98%_ and D_95%_ for PTV and GTV/CTV (*GTV for all cases except prostate; CTV in case of prostate) for the predicted (pr.) and re-optimized (re.) dose distributions. Dose normalized to the ideally achieved PTV encompassing dose (see sub-section “Comparison of DVH parameters and statistical analysis”)

For the target volume DVH parameters, the largest changes were found for D_98%_ and D_95%_ for the PTV and GTV/CTV when comparing the re-optimized with the predicted doses (Fig. 3). All PTV D_98%_ and D_95%_ median dose values increased significantly across all subgroups. More importantly, the GTV/CTV D_98%_ and D_95%_ values increased significantly, except for pancreas, where no significant difference was found (see Table 3). This means that the target volume dose coverage increased significantly in most cases when adapting. The largest median increases of PTV D_95%_ were found in liver, lung and lymph nodes. The largest median increases in GTV D_95%_ were also found in the same subgroups. Although a relatively large significant increase was found for PTV D_95%_ in pancreatic cases, no significant increase was found for the pancreas GTV D_98%_ and GTV D_95%_. Despite being statistically significant, smaller median increases in PTV D_98%_, PTV D_95%_, GTV/CTV D_98%_ and GTV/CTV D_95%_ were found for prostate, compared to the liver, lung and lymph node subgroups. All *p*-values and median percent increases are shown in Table 3.

**Fig. 4:**
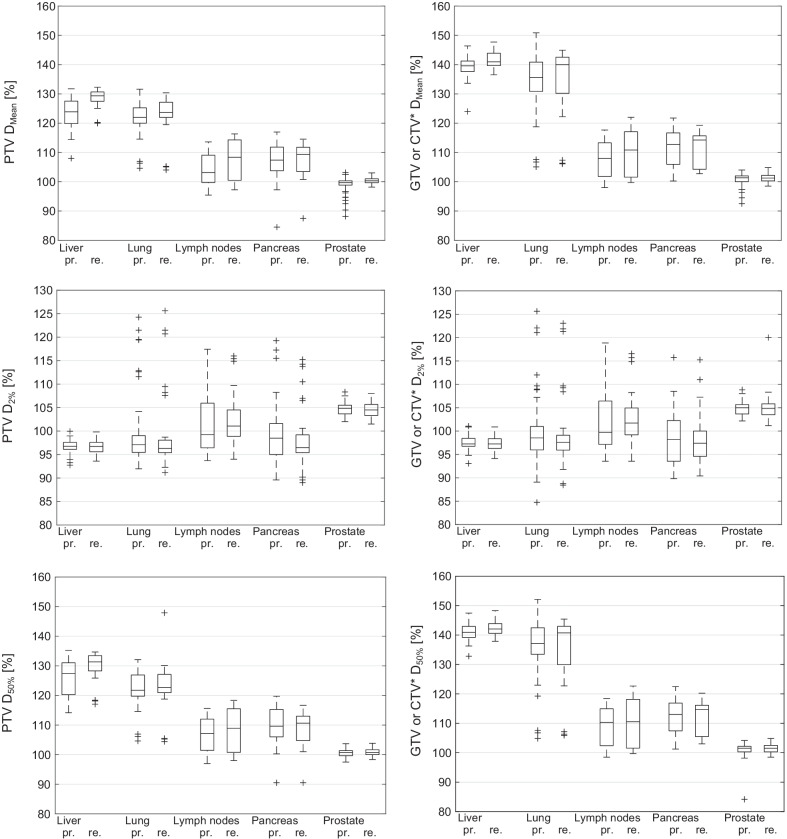
Boxplots for all subgroups of DVH target volume dose values D_50%_, D_2%_ and D_mean_ for PTV and GTV or CTV (*GTV for all cases except prostate; CTV in case of prostate) for the predicted (pr.) and re-optimized (re.) dose distributions. D_50%_ and D_mean_ normalized to the prescribed dose. D_2%_ normalized to the ideally achieved maximum dose (see sub-section “Comparison of DVH parameters and statistical analysis”)

Additional target dose volume parameters are shown in Fig. 4. The near maximum doses D_2%_ did not show significant changes in most cases. Significant but small median near maximum dose reductions inside the PTV or GTV were found for PTV D_2%_ (pancreas) and GTV D_2%_ (lung). When looking at the boxplots (Fig. 4), it can be seen that a few cases of the lung, lymph node and pancreas subgroups showed predicted near maximum doses, which exceeded 10% of the ideally achieved maximum dose (mostly outliers). A slight reduction of these high near maximum doses was achieved when adapting. After plan adaption, all non-outlier near-maximum values PTV D_2%_ and GTV D_2%_ exceeded the ideally achieved maximum dose by less than 10%. PTV and GTV/ CTV median (D_50%_) and mean (D_mean_) doses show an inverse behaviour. For these values, slight median increases were found in most cases, except for the pancreas subgroup, where no significant changes were found for PTV D_50%_, GTV D_50%_ and GTV D_mean_ (Table 3).

## Discussion

### Liver

For the liver subgroup, the initial predicted median PTV coverage (here PTV D_98%_ and PTV D_95%_) before plan adaption was worse compared to lung and prostate but was comparable to lymph nodes and pancreas. One reason might be that less complex shaped (sphere-like) targets, as small lung lesions (lung: mean V_PTV_ = 15.2 cm^3^ vs. liver: mean V_PTV_ = 38.4 cm^3^, see Table 2) or prostate targets, are easier to cover with default baseline plans. After plan adaption, a largely improved PTV coverage was found, which resulted in a close-to-ideal post-adaption PTV coverage, similar to that of the lung cases. With 93.3% of all fractions adapted, liver showed the highest portion of adapted plans (similar to pancreas with 92.0%). This means that the initial target volume coverage using the base plan was not ideal in almost every fraction. After plan adaption, PTV D_98%_ was > 97.0% of the PD in all fractions. Even though the initial median PTV coverage was worse compared to the lung cases, the initial GTV coverage was similar, which means that the PTV, designed for liver cases in breath hold technique, worked very well. When comparing the normalized percent values of Fig. 3 between these two (or any other) subgroups, especially for the GTV, one has to bear in mind that all liver cases had a stereotactic prescription to the 65% isodose, but only 8/10 lung cases had the same prescription. No significant change in the PTV or GTV near-maximum dose was found, just like for most other subgroups. Regarding OAR sparing, Fig. 2 shows a more or less symmetrical distribution around zero for liver, but with a significantly increased median OAR dose. This is because OAR dose limits were, on average, not fully reached prior to adaption. This tendency can also be seen when looking at the exposure changes of the three most frequently considered OARs (bowel, duodenum, stomach, see Table 4), where mean and median increases in dose for all these OARs were found for liver. During the optimization process, the OAR exposure was fully exploited and brought closer to the dose limits, in order to achieve a very good target coverage without violation of OAR constraint. With a mean number of 33 segments, adapted liver plans were simpler compared to those of lymph node, pancreas and prostate cases.

### Lung

For lung cases, the initial PTV coverage of the predicted base plan was better compared to liver, but could still be significantly improved and resulted in a near-optimal PTV coverage after plan adaption. The initial GTV coverage was similar to that of liver, but could still be significantly improved. With 84.4% of all fractions adapted, lung showed similar adaption rates as lymph node cases. Regarding OAR sparing and change of PTV near-maximum doses, similar findings were made as for the liver subgroup. The mean PTV volume of lung cases was the smallest of all subgroups (15.2 cm^3^) and so was the mean number of segments (26), indicating easy-to-adapt, simple treatment plans.

### Abdominal lymph nodes

For the abdominal lymph nodes subgroup, initial PTV coverage was not ideal but could be efficiently improved with online plan adaption, which was performed in 82.1% of fractions. As for to the liver and lung cases, a relatively large median increase (6.8%) in GTV D_98%_ was found without an increase in the near-maximum dose. The median OAR dose could be reduced significantly by − 4.5% (Table 3). A larger reduction of OAR dose was only observed in the pancreas subgroup. The lymph node treatment plans had an average number of 72 segments, which is similar to that of pancreas (70). This shows that lymph node treatment plans were usually highly modulated, similar to the pancreas plans.

### Pancreas

Plan adaptation resulted in a significant improvement in PTV near minimum dose. Regarding the GTV coverage, no significant improvement could be made on average, when performing online plan adaption. Although the average re-optimized (and even predicted) GTV coverage was acceptable, some cases were observed where sufficient GTV coverage could not be achieved, even after plan adaption, because sparing of OARs was prioritized. This can be seen in Fig. 3, when looking at the relatively large interval between the first and third quartiles compared to those of the abdominal lymph nodes and prostate cases. The large portion of adapted fractions (92.0%) indicates the need for plan adaptation to reduce OAR doses to meet clinically acceptable OAR dose levels. OAR doses could be reduced in more than 75.0% of all OAR constraints of all adapted fractions of the pancreas cases and the median OAR dose reduction was − 87.0% (Fig. 2). The V33_Gy_ of duodenum, stomach and bowel was reduced on average by more than 80% respectively (Table 4). Without online plan adaption, OAR dose limits would have been frequently violated.

### Prostate

For most prostate cases, the initial PTV and especially the CTV coverage were acceptable. In only a few fractions very insufficient initial target volume coverage was found (Fig. 3). For these fractions, large improvements in PTV and even CTV coverage were made when re-optimizing the plans. On average, no significant change of OAR exposure was achieved with online plan adaption. A mean of 54 segments were needed to achieve acceptable plans, which indicates plans of medium complexity. Only 69.4% of fractions were adapted.

### Comparison between tumor entities

All subgroups clearly benefited from online plan adaption in terms of improved PTV coverage. The improved target dose varied between the different tumor entities. To estimate the absolute dose changes of the target volumes achieved by online plan adaption, it is possible to multiply the total prescribed dose of any dose prescription (Table 1) with the corresponding median percent change of any DVH parameter of interest (Table 3) and with the percentage of adapted fractions of the corresponding patient subgroup (Table 2, line 1). Obviously, this formula can only provide a rough estimate of absolute dose changes and does not consider individual patients. Based on the underlying idea of the PTV target volume concept, one would assume that the GTV/CTV coverage physically achieved during dose application is of higher prognostic value than the PTV coverage, since the sole purpose of the PTV margins is to guarantee the GTV/CTV coverage with some degree of confidence. Under this assumption it is possible to identify subgroups that might benefit more from online plan adaption than others. In the present study, the three subgroups liver, lung, and abdominal lymph nodes had the greatest benefit from online plan adaption in terms of improved GTV coverage. After plan adaption, all three subgroups showed excellent GTV coverage (D_98%_ and D_95%_). In addition, a large portion of fractions (> 82%) required re-optimization in all three subgroups, indicating that most of these fractions can be significantly improved by online plan adaption. Since small values of GTV D_98%_ and D_95%_ indicate insufficient GTV coverage, these indices can be considered the most predictive (of all DVH parameters examined) in terms of improved tumor control probability (TCP) and possibly LC when comparing non-adapted and online adapted fractions. However, it remains unclear if the significant increases in GTV near-minimum and mean doses will translate into a detectable improved LC.

In addition to the significantly improved GTV coverage in the liver, lung and abdominal lymph nodes subgroups when using plan adaption, these subgroups might also largely benefit from the breath-hold and automated beam gating capabilities of the oMRgRT system, since no internal target volume concept is needed. An internal target volume concept would increase the total irradiated volume [51], especially for these cases, where breathing-related motion of target volumes can be frequently seen. In this study, the influence of beam-gating was not investigated and no motion range assessment was made for the target volumes. That is beyond the scope of this study.

It was found for pancreas, that the plan adaption capabilities were largely used to reduce OAR doses to an acceptable level. Although the re-optimized GTV coverage was acceptable in most cases, the improvement in GTV coverage via online plan adaption was not as large as in the other subgroups, as OAR sparing was prioritized. In contrast, in the prostate subgroup, only 69.4% of all fractions were considered for re-optimization. For most of these fractions, the initial CTV coverage was quite good but could still be slightly improved when adapted. Ultimately, few fractions were found, where the initial PTV and CTV coverage was unacceptable. For these fractions, online re-optimization resulted in an excellent CTV coverage. In summary, for prostate cases, the benefit of online re-optimization was found to be not as systematic as for the liver, lung, abdominal lymph nodes and pancreas cases. In each subgroup, at least one of the primary dosimetric endpoints defined under “Methods” (significant increase in GTV/CTV near-minimum and mean dose, and significantly reduced OAR exposure) was achieved (Table 3). Except for pancreas, all other subgroups met all primary endpoints related to improved GTV/CTV dose.

To the best of our knowledge, up to now, no attempt has been made to quantify the influence of MR-guided online plan adaption on DVH-related parameters and systemically compare the results between multiple subgroups with different tumor entities typically treated on integrated MR-Linac systems. Our intent was to provide information for a more informed decision making when assigning patients to the (still very limited access) MR-Linac. Henke et al. [40] analyzed 81 online adapted fractions (20 patients) in a patient cohort of mixed oligometastatic or unresectable abdominal malignancies (hepatic lesions, adrenal metastasis, pancreatic adenocarcinoma and lymph node metastases). The overall adaption rate (83.5%) was comparable to our study, although less liver fractions (66.0%) were adapted compared to our liver subgroup (93.3%). Similar to the present study, several fractions among the pancreas cases were found, where GTV dose de-escalation was necessary due to OAR proximity of the tumor. Regnery et al. [52] prospectively compared predicted versus adapted dose distributions in 154 online adapted fractions in 21 lung tumor patients. The higher adaption rate of 93.3% compared to 84.4% in the current study can be explained with the large number of ultracentral lung tumors in the cohort of Regnery et al., where OAR violations are more likely to occur due to adjacency OARs. Regnery et al. found a large increase in the minimum biologically effective dose (BED) of the PTV and a moderate increase in the minimum BED of the GTV. We observed the same tendency when considering PTV and GTV D_98%_ or D_95%_. In the same study only small increases in mean BED inside the PTV and GTV were found, which is also in accordance with our findings of PTV and GTV D_mean_. El Bared et al. [35] evaluated the dosimetric benefits in 10 patients treated for unresectable pancreatic cancer on a cobalt-60, 0.35 T MRI system when performing online plan adaption and reported outcome. Although comparability to our study is limited due to a fundamental discrepancy in technical design and beam quality (tri-source cobalt-60 vs. 6 MV flattening filter free Linac), El Bared also found improved PTV coverage when performing online plan adaption. However, the influence on GTV coverage was not evaluated. Placidi et al. [37] found a similar trend of improved PTV coverage in 8 pancreatic cancer patients also treated on the cobalt-60 system and similarly reported increased CTV dose after plan adaption. The influence on GTV coverage was not evaluated. Mayinger et al. [53] analyzed online adapted treatment plans of 15 patients with liver metastases and found improved PTV coverage in cases where the target volume was in close proximity to OARs. The influence on GTV coverage was not evaluated in detail. In the present study, we did not stratify patients for adjacency of OARs, but when looking at the PTV mean and near-minimum doses, we also found a significant increase after plan adaption in liver cases. Padgett et al. [43] artificially created adapted plans for 10 patients with liver cancers on the cobalt-60 system and compared the results to the non-adapted plans and also found improved PTV and GTV coverage as well as a reduced number of OAR violations (duodenum, bowel and stomach) after plan adaption. We observed the same trend regarding target volumes and reported small mean and median dose increases for OARs like duodenum, bowel and stomach. This is no contradiction to the findings of Padgett et al. since OAR sparing was prioritized over target volume coverage in our study and no hard OAR constraints were violated during plan adaption.

## Conclusions

All subgroups clearly benefited from online plan adaption in terms of improved PTV coverage. Moreover, for the liver, lung and abdominal lymph node cases, a systematic improvement in GTV coverage was found, resulting in excellent target coverage after re-optimization in most fractions. In combination with the breath-hold-based technique, these subgroups can fully exploit the potential of oMRgRT systems. In the pancreatic cancer subgroup, online plan adaption resulted in largely decreased OAR doses but the target coverage could not always be improved due to the limiting OAR constraints. While many fractions of the prostate subgroup could, in theory, also be effectively treated without plan adaption, most fractions still showed improved PTV coverage and few fractions even showed large CTV coverage improvements after online plan re-optimization.

## Data Availability

Not applicable.

## References

[1] Hunt A, Hansen VN, Oelfke U, Nill S, Hafeez S (2018). Adaptive radiotherapy enabled by MRI guidance. Clin Oncol.

[2] Bohoudi O, Bruynzeel AME, Senan S, Cuijpers JP, Slotman BJ, Lagerwaard FJ, Palacios MA (2017). Fast and robust online adaptive planning in stereotactic MR-guided adaptive radiation therapy (SMART) for pancreatic cancer. Radiother Oncol.

[3] Heerkens HD, van Vulpen M, van den Berg CAT, Tijssen RHN, Crijns SPM, Molenaar IQ (2014). MRI-based tumor motion characterization and gating schemes for radiation therapy of pancreatic cancer. Radiother Oncol.

[4] Heijkoop ST, Langerak TR, Quint S, Bondar L, Mens JWM, Heijmen BJM, Hoogeman MS (2014). Clinical implementation of an online adaptive plan-of-the-day protocol for nonrigid motion management in locally advanced cervical cancer IMRT. Int J Radiat Oncol Biol Phys.

[5] Liu F, Erickson B, Peng C, Li XA (2012). Characterization and management of interfractional anatomic changes for pancreatic cancer radiotherapy. Int J Radiat Oncol Biol Phys.

[6] McPartlin AJ, Li XA, Kershaw LE, Heide U, Kerkmeijer L, Lawton C (2016). MRI-guided prostate adaptive radiotherapy—a systematic review. Radiother Oncol.

[7] Pathmanathan AU, van As NJ, Kerkmeijer LGW, Christodouleas J, Lawton CAF, Vesprini D (2018). Magnetic resonance imaging-guided adaptive radiation therapy: a “game changer” for prostate treatment?. Int J Radiat Oncol Biol Phys.

[8] Ahunbay EE, Peng C, Chen G-P, Narayanan S, Yu C, Lawton C, Li XA (2008). An on-line replanning scheme for interfractional variations. Med Phys.

[9] Ahunbay EE, Peng C, Holmes S, Godley A, Lawton C, Li XA (2010). Online adaptive replanning method for prostate radiotherapy. Int J Radiat Oncol Biol Phys.

[10] Foroudi F, Wong J, Kron T, Rolfo A, Haworth A, Roxby P (2011). Online adaptive radiotherapy for muscle-invasive bladder cancer: results of a pilot study. Int J Radiat Oncol Biol Phys.

[11] Li Y, Hoisak JDP, Li N, Jiang C, Tian Z, Gautier Q (2015). Dosimetric benefit of adaptive re-planning in pancreatic cancer stereotactic body radiotherapy. Med Dosim.

[12] Mohan R, Zhang X, Wang H, Kang Y, Wang X, Liu H (2005). Use of deformed intensity distributions for on-line modification of image-guided IMRT to account for interfractional anatomic changes. Int J Radiat Oncol Biol Phys.

[13] Castelli J, Simon A, Louvel G, Henry O, Chajon E, Nassef M (2015). Impact of head and neck cancer adaptive radiotherapy to spare the parotid glands and decrease the risk of xerostomia. Radiat Oncol.

[14] Guckenberger M, Wilbert J, Richter A, Baier K, Flentje M (2011). Potential of adaptive radiotherapy to escalate the radiation dose in combined radiochemotherapy for locally advanced non-small cell lung cancer. Int J Radiat Oncol Biol Phys.

[15] Henke LE, Kashani R, Hilliard J, DeWees TA, Curcuru A, Przybysz D (2018). In silico trial of MR-guided midtreatment adaptive planning for hypofractionated stereotactic radiation therapy in centrally located thoracic tumors. Int J Radiat Oncol Biol Phys.

[16] de Jong R, Visser J, Crama KF, van Wieringen N, Wiersma J, Geijsen ED, Bel A (2020). Dosimetric benefit of an adaptive treatment by means of plan selection for rectal cancer patients in both short and long course radiation therapy. Radiat Oncol.

[17] Mohamed ASR, Bahig H, Aristophanous M, Blanchard P, Kamal M, Ding Y (2018). Prospective in silico study of the feasibility and dosimetric advantages of MRI-guided dose adaptation for human papillomavirus positive oropharyngeal cancer patients compared with standard IMRT. Clin Transl Radiat Oncol.

[18] Møller DS, Holt MI, Alber M, Tvilum M, Khalil AA, Knap MM, Hoffmann L (2016). Adaptive radiotherapy for advanced lung cancer ensures target coverage and decreases lung dose. Radiother Oncol.

[19] Nijkamp J, Pos FJ, Nuver TT, de Jong R, Remeijer P, Sonke J-J, Lebesque JV (2008). Adaptive radiotherapy for prostate cancer using kilovoltage cone-beam computed tomography: first clinical results. Int J Radiat Oncol Biol Phys.

[20] Oh S, Stewart J, Moseley J, Kelly V, Lim K, Xie J (2014). Hybrid adaptive radiotherapy with on-line MRI in cervix cancer IMRT. Radiother Oncol.

[21] van de Schoot AJAJ, de Boer P, Visser J, Stalpers LJA, Rasch CRN, Bel A (2017). Dosimetric advantages of a clinical daily adaptive plan selection strategy compared with a non-adaptive strategy in cervical cancer radiation therapy. Acta Oncol.

[22] Yoon SW, Lin H, Alonso-Basanta M, Anderson N, Apinorasethkul O, Cooper K (2020). Initial evaluation of a novel cone-beam CT-based semi-automated online adaptive radiotherapy system for head and neck cancer treatment—a timing and automation quality study. Cureus.

[23] Inc., Viewray. First patients treated with ViewRay's MRIdian Linac System at Henry Ford Health System. 2017.

[24] Corradini S, Alongi F, Andratschke N, Belka C, Boldrini L, Cellini F (2019). MR-guidance in clinical reality: current treatment challenges and future perspectives. Radiat Oncol.

[25] Acharya S, Fischer-Valuck BW, Kashani R, Parikh P, Yang D, Zhao T (2016). Online magnetic resonance image guided adaptive radiation therapy: first clinical applications. Int J Radiat Oncol Biol Phys.

[26] Fischer-Valuck BW, Henke L, Green O, Kashani R, Acharya S, Bradley JD (2017). Two-and-a-half-year clinical experience with the world's first magnetic resonance image guided radiation therapy system. Adv Radiat Oncol.

[27] Güngör G, Serbez İ, Temur B, Gür G, Kayalılar N, Mustafayev TZ (2020). Time analysis of online adaptive magnetic resonance-guided radiation therapy workflow according to anatomical sites. Pract Radiat Oncol.

[28] Lagerwaard F, Bohoudi O, Tetar S, Admiraal MA, Rosario TS, Bruynzeel A (2018). Combined inter- and intrafractional plan adaptation using fraction partitioning in magnetic resonance-guided radiotherapy delivery. Cureus.

[29] Paulson ES, Ahunbay E, Chen X, Mickevicius NJ, Chen G-P, Schultz C (2020). 4D-MRI driven MR-guided online adaptive radiotherapy for abdominal stereotactic body radiation therapy on a high field MR-Linac: implementation and initial clinical experience. Clin Transl Radiat Oncol.

[30] Raaymakers BW, Jürgenliemk-Schulz IM, Bol GH, Glitzner M, Kotte ANTJ, van Asselen B (2017). First patients treated with a 1.5 T MRI-Linac: clinical proof of concept of a high-precision, high-field MRI guided radiotherapy treatment. Phys Med Biol.

[31] Tetar SU, Bruynzeel AM, Lagerwaard FJ, Slotman BJ, Bohoudi O, Palacios MA (2019). Clinical implementation of magnetic resonance imaging guided adaptive radiotherapy for localized prostate cancer. Phys Imaging Radiat Oncol.

[32] Bruynzeel AME, Tetar SU, Oei SS, Senan S, Haasbeek CJA, Spoelstra FOB (2019). A prospective single-arm phase II study of stereotactic magnetic-resonance-guided adaptive radiotherapy for prostate cancer: early toxicity results. Int J Radiat Oncol Biol Phys.

[33] Nicosia L, Sicignano G, Rigo M, Figlia V, Cuccia F, de Simone A (2020). Daily dosimetric variation between image-guided volumetric modulated arc radiotherapy and MR-guided daily adaptive radiotherapy for prostate cancer stereotactic body radiotherapy. Acta Oncol.

[34] Menten MJ, Wetscherek A, Fast MF (2017). MRI-guided lung SBRT: present and future developments. Phys Med.

[35] El-Bared N, Portelance L, Spieler BO, Kwon D, Padgett KR, Brown KM, Mellon EA (2019). Dosimetric benefits and practical pitfalls of daily online adaptive MRI-guided stereotactic radiation therapy for pancreatic cancer. Pract Radiat Oncol.

[36] Hassanzadeh C, Rudra S, Bommireddy A, Hawkins WG, Wang-Gillam A, Fields RC (2020). Ablative five-fraction stereotactic body radiation therapy for inoperable pancreatic cancer using online MR-guided adaptation. Adv Radiat Oncol.

[37] Placidi L, Romano A, Chiloiro G, Cusumano D, Boldrini L, Cellini F (2020). On-line adaptive MR guided radiotherapy for locally advanced pancreatic cancer: clinical and dosimetric considerations. Techn Innov Patient Support Radiat Oncol.

[38] Rudra S, Jiang N, Rosenberg SA, Olsen JR, Roach MC, Wan L (2019). Using adaptive magnetic resonance image-guided radiation therapy for treatment of inoperable pancreatic cancer. Cancer Med.

[39] Kerkhof EM, Raaymakers BW, van der Heide UA, van de Bunt L, Jürgenliemk-Schulz IM, Lagendijk JJW (2008). Online MRI guidance for healthy tissue sparing in patients with cervical cancer: an IMRT planning study. Radiother Oncol.

[40] Henke L, Kashani R, Robinson C, Curcuru A, DeWees T, Bradley J (2018). Phase I trial of stereotactic MR-guided online adaptive radiation therapy (SMART) for the treatment of oligometastatic or unresectable primary malignancies of the abdomen. Radiother Oncol.

[41] Henke LE, Olsen JR, Contreras JA, Curcuru A, DeWees TA, Green OL (2019). Stereotactic MR-guided online adaptive radiation therapy (SMART) for ultracentral thorax malignancies: results of a phase 1 trial. Adv Radiat Oncol.

[42] Mittauer KE, Hill PM, Geurts MW, de Costa A-M, Kimple RJ, Bassetti MF, Bayouth JE (2019). STAT-ART: the promise and practice of a rapid palliative single session of MR-guided online adaptive radiotherapy (ART). Front Oncol.

[43] Padgett KR, Simpson G, Asher D, Portelance L, Bossart E, Dogan N (2020). Assessment of online adaptive MR-guided stereotactic body radiotherapy of liver cancers. Phys Med.

[44] Palacios MA, Bohoudi O, Bruynzeel AM, van Sörsen de Koste JR, Cobussen P, Slotman BJ (2018). Role of daily plan adaptation in MR-guided stereotactic ablative radiation therapy for adrenal metastases. Int J Radiat Oncol Biol Phys.

[45] Vestergaard A, Hafeez S, Muren LP, Nill S, Høyer M, Hansen VN (2016). The potential of MRI-guided online adaptive re-optimisation in radiotherapy of urinary bladder cancer. Radiother Oncol.

[46] van Timmeren JE, Chamberlain M, Krayenbuehl J, Wilke L, Ehrbar S, Bogowicz M (2020). Treatment plan quality during online adaptive re-planning. Radiat Oncol.

[47] Klüter S (2019). Technical design and concept of a 0.35 T MR-Linac. Clin Transl Radiat Oncol.

[48] Ahunbay EE, Li XA (2015). Gradient maintenance: a new algorithm for fast online replanning. Med Phys.

[49] Andratschke N, Parys A, Stadtfeld S, Wurster S, Huttenlocher S, Imhoff D (2016). Clinical results of mean GTV dose optimized robotic guided SBRT for liver metastases. Radiat Oncol.

[50] Baumann R, Chan MKH, Pyschny F, Stera S, Malzkuhn B, Wurster S (2018). Clinical results of mean GTV dose optimized robotic-guided stereotactic body radiation therapy for lung tumors. Front Oncol.

[51] Finazzi T, Palacios MA, Haasbeek CJA, Admiraal MA, Spoelstra FOB, Bruynzeel AME (2020). Stereotactic MR-guided adaptive radiation therapy for peripheral lung tumors. Radiother Oncol.

[52] Regnery S, Buchele C, Weykamp F, Pohl M, Hoegen P, Eichkorn T (2022). Adaptive MR-guided stereotactic radiotherapy is beneficial for ablative treatment of lung tumors in high-risk locations. Front Oncol.

[53] Mayinger M, Ludwig R, Christ SM, Dal Bello R, Ryu A, Weitkamp N (2021). Benefit of replanning in MR-guided online adaptive radiation therapy in the treatment of liver metastasis. Radiat Oncol.

